# Adult Neural Stem Cells: Redefining the Physio- and Pathology of the CNS

**Published:** 2008-03

**Authors:** Philippe Taupin

**Affiliations:** 1 *National Neuroscience Institute, Singapore;*; 2 *National University of Singapore, Singapore;*; 3 *Nanyang Technological University, Singapore*

**Keywords:** neurogenesis, stem cells, pharmacology, development, plasticity, regeneration, neurological diseases and disorders

## Abstract

Stem cells are the “building blocks” of the body; they are self-renewing undifferentiated cells that give rise to the specialized cells of the tissues. In adult, stem cells are multipotent, they contribute to homeostasis of the tissues and regeneration after injury. Until recently, it was believed that the adult brain was devoid of stem cells, hence unable to make new neurons and regenerate. The recent confirmation that neurogenesis occurs in the adult brain and neural stem cells (NSCs) reside in the adult central nervous system (CNS) suggests that the adult brain has the potential to regenerate and may be amenable to repair. The advent of adult neurogenesis and NSC research will redefine our understanding of the physio- and pathology of the nervous system, and provide new avenues and opportunities to treat a broad range of neurological diseases, disorders and injuries. Adult NSC-based therapies will involve cellular therapy, but also pharmacology.

## INTRODUCTION

Seminal studies in the 60s, conducted by Altman and Das, were the first to report the generation of new neuronal cells in the adult mammalian brain. The authors reported, using [3H]-thymidine autoradiographic labeling, the generation of new neuronal cells in the dentate gyrus (DG), and cell proliferation in the ventricular zone, migration and persisting neurogenesis in the olfactory bulb (OB) of adult rodents (1, 2). With the advent of new procedures for labeling dividing cells in the CNS, like bromodeoxyuridine (BrdU), retroviral labelings and magnetic resonance nuclear, new studies have since confirmed that neurogenesis occurs in the adult mammalian brain, primarily in two regions: the DG of the hippocampus and the subventricular zone (SVZ), in several species (3-5), including human (6-8). The advent of adult neurogenesis and NCS research has tremendous implications for our understanding of the physio- and pathology of the CNS, as well as for therapy. Over the past decades, significant progresses have been made in the field of research. However, there is much debates, controversies and questions to be answered.

## ADULT NEUROGENESIS AND NEURAL STEM CELLS

### Neurogenesis in the adult brain

In the DG, newly generated neuronal cells in the subgranular zone (SGZ) migrate to the granular layer, where they differentiate into mature neuronal cells, and extend axonal projections to the CA3 region. In the SVZ, cells are generated in the anterior part of the SVZ and migrate to the OB, where they differentiate into interneurons (9). Newly generated neuronal cells establish functional connections with neighboring cells (5, 10), particularly GABAergic innervations in the DG, soon after their migration is completed (11). Approximately 9,000 new neuronal cells or 0.1% of the granule cell population are generated per day in the DG, and 65.3-76.9% of the bulbar neurons are replaced during a 6 weeks period, in young adult rodents (12-14). Among them, a significant proportion undergoes programmed cell death rather than achieving maturity (14-16). The newly generated neuronal cells that survived to maturity may be very stable, and may permanently replace cells born during development, as adult-generated neuronal cells have been reported to survive for extended period of time (17, 18), at least 2 years in human DG (6).

Neurogenesis may also occur, albeit at lower levels, in other areas of the adult mammalian brain, like the Ammon’s horn CA1, neocortex, substantia nigra, and the 3rd ventricle in certain species (19-22). However, some of these reports have been contradicted by other studies, and need to be further evaluated (23-25).

### Origin of newly generated neuronal cells in the adult brain

The origin of newly generated neuronal cells in the adult brain remains the source of controversies and to be fully determined. One theory contends that they originate from differentiated ependymal cells in the lateral ventricle, while another contends that they originate from astrocyte-like cells (9). A glial origin for adult generated neuronal cells in the SVZ and SGZ has received further support (26, 27). As for the origin of newly generated neuronal cells in other areas of the brain, it remains to be determined (16, 25).

It is postulated that newly generated neuronal cells originate from residual stem cells in the adult brain. NSCs are the self-renewing, multipotent cells that generate neurons, astrocytes and oligodendrocytes of the nervous system. In support of this contention, self-renewing, multipotent neural progenitor and stem cells have been isolated and characterized *in vitro* from various areas of the adult CNS, neurogenic and non-neurogenic, including the spinal cord, suggesting that neural progenitor and stem cells may reside throughout the CNS (9).

There are currently no specific markers of adult NSCs. The intermediate neurofilament nestin, the transcription factors sox-2, oct-3/4, and the RNA binding protein Musashi 1 are markers for neural progenitor and stem cells, but also label population of glial cells (28-34), further fueling the controversies and debates over the origin of newly generated neuronal cells in the adult brain.

### Adult neurogenesis is modulated

The rate of neurogenesis in the adult rodent DG and SVZ is modulated by various conditions, like environmental stimuli, physio- and pathological processes, trophic factors/cytokines and drugs (35, 36). Environmental enrichment promotes the survival of newly generated neuronal cells in the DG (12). Voluntary running stimulates the generation of newly generated neuronal cells in the DG, but not the SVZ. Learning and memory increases neurogenesis in the adult DG. Stress, neuroinflammation and aging decrease neurogenesis in the adult DG. In the diseased brain and after injuries to the CNS, like in Huntington’s disease (HD) and after cerebral strokes, neurogenesis is stimulated in the neurogenic areas, and new neuronal cells are generated at the sites of injuries, where they replace some of the degenerated nerve cells (36). Cell tracking studies revealed that newly generated neuronal cells at sites of injuries originates from the SVZ. Newly generated neuronal cells migrate partially through the RMS to the degenerated areas. It is estimated that 0.2% of the degenerated nerve cells are replaced in the striatum after focal ischemia (37). Epidermal growth factor (EGF) stimulates the proliferation of neural progenitor cells in the adult rat SVZ (38, 39). Insulin-like growth factor-I (IGF-I) stimulates neurogenesis in the adult rat DG (40). Galantamine and memantine, two drugs used to treat Alzheimer’s disease (AD), increase neurogenesis in the adult DG and SVZ (41). Chronic administration of antidepressants, like the selective serotonin reuptake inhibitor (SSRI) fluoxetine, increases neurogenesis in the adult DG, but not the SVZ (42-44).

The modulation of adult neurogenesis suggests that it may be involved in the physio- and pathology of the nervous system, as well as in mediating drugs activity.

Some studies have shown that cell death stimulates the proliferation of neural progenitor cells in the adult hippocampus (45), while others that the mitotic rate is regulated by the number of available progenitor cells, rather than by cell death (46, 47). EGF and basic fibroblast growth factor were the first mitogens to be identified for neural progenitor and stem cells *in vitro* (48, 49). Other factors present in conditioned medium, like the glycosylated form of the protease inhibitor cystatin C (CCg), are required for the proliferation of self-renewing, multipotent NSCs from single cells *in vitro* (50). The regulation of neurogenesis has been reported to be mediated by the estrogen-receptor (51), the activity of the hypothalamic-pituitary-adrenal axis (52), as well as the IGF pathway (53), but not the glutamatergic pathway (54). However, most the mechanisms underlying adult neurogenesis and its modulation are yet to be uncovered.

### Limits of BrdU labeling for studying neurogenesis

The modulation of neurogenesis and its quantification have been subject of debates, partly due to the use of BrdU labeling as a method of assessment. As BrdU crosses the blood-brain barrier, it is generally administered intraperiteonally. Activity, like exercise, but also the effect of various physio- and pathological conditions affect the cerebral flow, metabolism and permeability of the blood-brain barrier. This may affect the bio-availability of BrdU in the brain. The variation of BrdU quantification observed in these conditions would then reflect the change in BrdU uptake by the cells, rather than the modulation neurogenesis (55).

With regard to the quantification of neurogenesis with BrdU, one study suggests that the standard concentration used to assess neurogenesis (50-100 mg/kg body weight in rodents, intraperitoneal injection) may not label all the dividing cells, whereas another study reports that it does (55, 56). Further systematic studies on BrdU labeling in the CNS are thus needed to precise the conditions in which BrdU can be used for studying neurogenesis. The use of BrdU to study neurogenesis carries other limitations, like labeling of DNA repair, abortive cell cycle reentry and gene duplication. Other strategies are therefore necessary to make educated conclusions with regard to adult neurogenesis when using BrdU labeling, like the study of markers of the cell cycle and the use of retroviruses (55).

## CELLULAR THERAPY AND PHARMACOLOGY

The evidences that neurogenesis occurs in the adult brain and NSCs reside in the adult CNS provide new avenues and opportunities for cellular therapy. Cell therapeutic intervention may involve the stimulation of endogenous or the transplantation of neural progenitor and stem cells of the adult CNS.

### Stimulation of endogenous neural progenitor and stem cells of the adult CNS

The administration of EGF and IGF-I, have been reported to promote neurogenesis (38-40). Hence, the administration of trophic factors/cytokines represents a valid strategy to promote regeneration and repair the nervous system, by stimulating endogenous neural progenitor and stem cells locally. New neuronal cells are generated at sites of degeneration in the diseased brain and after CNS injuries, like in HD and in experimental models of cerebral strokes (36, 37). They originate from the SVZ and migrate to the sites of degeneration, partially through the RMS. Hence, strategies to promote regeneration and repair may aim at stimulating SVZ neurogenesis. To this aim, the intracerebroventricular administration of trophic factors provides may represent a strategy to promote SVZ neurogenesis in the diseased or injured nervous system (38, 39).

### Transplantation of adult-derived neural progenitor and stem cells

Neural progenitor and stem cells can be isolated from the adult brain and cultured *in vitro* from various regions of the CNS, including from human biopsies and post mortem tissues (57). The transplantation of adult-derived neural progenitor and stem cells provide an opportunity for cellular therapy. Intracerebral transplantation aims at replacing unhealthy or damaged tissues and is particularly suitable for diseases where neurodegeneration is limited to discrete regions, like in Parkinson’s disease. Such strategy may not be applicable for diseases where the degeneration is widespread, like AD, HD and multiple sclerosis. Neural progenitor and stem cells, administered intravenously, migrate to diseased and injured sites of the brain (58, 59). Systemic injection of adult-derived neural progenitor and stem cells may represent an alternative strategy for the treatment of neurological diseases and injuries, where the degeneration is widespread.

### Pharmacology

The modulation of adult neurogenesis, by drugs used to treat AD and depression, suggests that adult neurogenesis may be involved in mediating the activities of drugs used to treat neurological diseases and disorders (41, 44, 60). This further suggests that adult neurogenesis may be involved in the etiology and pathogenesis of these diseases. The notion that the activity of these drugs may act on or be mediated through adult neurogenesis offers new opportunities to treat neurological diseases and disorders, particularly AD and depression. However, it remains to determine the contribution of adult neurogenesis to neurological diseases and disorders, as well as the mechanisms of activity of drugs used to treat these diseases on adult neurogenesis.

In all, adult neurogenesis and neural stem cells offer promising strategies to treat a broad range of neurological diseases, disorders and injuries. Among them, the pharmacological approach opens new therapeutic perspectives, but also the opportunity to devise new drugs, potentially more potent to treat neurological diseases and disorders.

## THE FUTURE OF ADULT NSCS: REDEFINING THE PHYSIO- AND PATHOLOGY OF THE NERVOUS SYSTEM

Newly generated neuronal cells represent a small fraction of nerve cells in the adult brain. But data presented above suggest that their relevance to CNS physio- and pathology, as significant, although yet to be uncovered. One of the key in our understanding of the biology of adult neurogenesis and NSCs will be to determine the involvement and relative contribution of adult NSCs, relative to the preexisting network, in the functioning of the nervous system. This will lead to redefine our knowledge of the CNS, from its development, plasticity, to its physio- and pathology.

**Figure 1:**
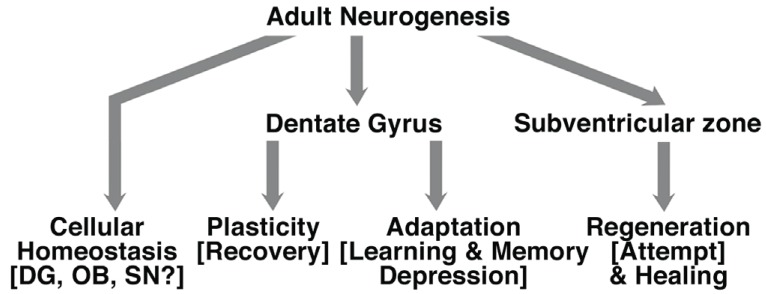
Involvement of adult neurogenesis in broad range of physio- and pathological processes. Adult neurogenesis is modulated in a broad range of physio- and pathological processes, like learning and memory, Alzheimer’s disease, cerebral strokes. However, the contribution of adult neurogenesis to this processes and hippocampal physiology remains to be determined. Adult neurogenesis may be involved in homeostasis of the tissue, neuroadaptative processes, plasticity and neuroregeneration.

Adult neurogenesis and NSCs may be involved in broad range of physio- and pathological processes (Fig. 1).

### Learning and memory

The function(s) of adult neurogenesis has been the source of intense research and debates. Evidences suggest that newly generated neuronal cells participate to processes like learning and memory (61, 62). The involvement of adult neurogenesis in learning and memory has been challenged by other studies. Increased hippocampal neurogenesis has been observed without improvement of learning and memory performances, in the Morris water maze test, in mice selectively bred for high levels of wheel running (63). Therefore the function of newly generated neuronal cells in the adult brain remains to be determined.

### Homeostasis of the tissue

The total number of neurons does not dramatically increase, and cell death is an established process in that adult brain (14, 15, 16). Newly generated neuronal cell may contribute to homeostasis of the adult brain. Neurogenesis occurs in discrete areas of the adult brain. This suggests that homeostasis of the tissue is limited in the adult brain. It remains to understand and determine the molecular, cellular and physiological underlying the occurrence of neurogenesis in discrete regions of the adult brain. Neurogenic niches have been described in the adult brain, and may hold the molecular and cellular cues to such phenomenon (64-67). On the physiological level, since environmental enrichment promotes adult neurogenesis and standard laboratory living conditions do not represent physiological environment, neurogenesis may occur more broadly, at low level - that would remain undetected-, in the adult brain of mammals (55). However, such hypothesis remains to be proven.

### Neuroadaptative process

The increase of neurogenesis in diseases, disorders, and after injuries might serve a neuroadaptative process. Patients with neurological diseases, like AD, epilepsy and Parkinson’s disease, but also recovering from stroke and injury, are at greater risk of depression (68-70) and present memory impairment (71, 72). Since learning and memory, depression are associated with hippocampal neurogenesis (60-62), the depressive episode and learning impairments in patients suffering from neurological diseases, or disorders may contribute to the regulation of neurogenesis, in an additive, or cooperative manner with the disorder. Therefore, modulation of neurogenesis in the hippocampus might be an attempt by the CNS to compensate for other neuronal functions associated with the disease, like depression, and learning and memory impairments.

### Plasticity

The increase in neurogenesis would also be a factor contributing to the plasticity of the CNS, and particularly related to the recovery in the CNS after injury. After cerebral strokes and traumatic brain injuries, there is a striking amount of neurological recovery in the following months and years, despite often-permanent structural damage (73). Though the mechanisms underlying such recovery are not fully understood, properties of plasticity of the CNS, like the reorganization of the pre-existing network and axonal sprouting have been implicated in the recovery (74). Particularly, reorganization of the contra-lateral hemisphere has been involved in plasticity after brain injury (73). Neurogenesis is increased bilaterally in the DG and the SVZ after cerebral strokes and traumatic brain injuries. The bilateral increase in neurogenesis would a factor contributing to the plasticity related recovery in the CNS, and particularly after injury (75).

### Neuroregeneration

The generation of newly generated neuronal cells at the sites of injury could represent a regenerative attempt by the CNS. In the diseased brain and after injuries to the CNS, new neuronal cells are generated at the sites of degeneration, where they replace some of the lost nerves cells (36, 37). Hence there is no functional recovery. The generation of new neuronal cells at the sites of injury could represent an attempt by the CNS to regenerate following injury. Several hypotheses can explain the lack of recovery of the CNS after injury. The number of new neurons generated may be too low to compensate for the neuronal loss -0.2% of the degenerated nerve cells in the striatum after focal ischemia- (37). The neuronal cells that are produced are non-functional because they do not develop into fully mature neurons, because they do not develop into the right type of neurons, or because they are incapable of integrating into the surviving brain circuitry.

## THE PROMISE OF ADULT NEURAL STEM CELLS

The promise of adult NSCs lie also in our ability to bring adult NSC research to therapy. Because of their potential to generate the main phenotype of the CNS, NSCs hold the promise to cure a broad range of CNS diseases and injuries. The confirmation that neurogenesis occurs in the adult brain and NSCs reside in the adult CNS opens new avenues and opportunities for the treatment of neurological diseases, disorders and injuries; adult NSC-based therapy may involve cellular therapy and pharmacology. Interestingly, the potential to isolate neural progenitor and stem cells from non-degenerated brain areas from the patient himself would provide an autologous source of transplantable neural progenitor and stem cells. However such strategy would involve invasive surgery and the destruction of healthy brain tissue, a limiting factor for its clinical application.

## CONCLUSION

The confirmation that neurogenesis occurs in the adult brain and NSCs reside in the adult CNS, in mammals, has tremendous implications for our understanding of brain development, physio-, pathology and therapy. The promise of adult neurogenesis and NSC research lies in our ability to bring NSC research to therapy. To this aim, the pharmacology of adult neurogenesis offers new perspectives to treat neurological diseases and disorders. The future of adult neurogenesis lies in redefining our understanding and knowledge of the development, physio- and pathology of the nervous system. Significant advances have already been made in just the past decades. However, many questions remain to be answered, and debates and controversies sorted-out.
